# Socioeconomic factors associated with poor patient-reported outcomes of 17,478 patients after a distal radial fracture

**DOI:** 10.1177/17531934241293426

**Published:** 2024-11-02

**Authors:** Hugo Jakobsson, Michael Möller, Yang Cao, Eva Lundqvist, Per Wretenberg, Marcus Sagerfors

**Affiliations:** 1 Department of Hand and Orthopedic Surgery, Faculty of Medicine and Health, Örebro University, Örebro, Sweden; 2 Institute of Clinical Sciences, Sahlgrenska Academy, Gothenburg University, Gothenburg, Sweden; 3 Clinical Epidemiology and Biostatistics, School of Medical Sciences, Faculty of Medicine and Health, Örebro University, Örebro, Sweden; 4 Unit of Integrative Epidemiology, Institute of Environmental Medicine, Karolinska Institute, Stockholm, Sweden

**Keywords:** Comorbidity, country of birth, distal radial fracture, Short Musculoskeletal Function Assessment, socioeconomic factors, wrist fracture

## Abstract

This study aimed to investigate the association of socioeconomic factors, country of birth and comorbidities with poor patient-reported outcome 1 year after a distal radial fracture. The patient population was obtained from the Swedish Fracture Register. In the study, 17,468 patients 18 years or older were included. Poor outcome was the dependent variable in a multivariate logistic regression analysis. The factors with the strongest association with poor outcome were country of birth outside the European Union (odds ratio (OR) = 2.28; 95% CI = 1.91–2.73), high-energy trauma mechanism (OR = 1.76; 95% CI = 1.46–2.12), a history of anxiety or depression (OR = 1.46; 95% CI = 1.26–1.70), and a Charlson comorbidity index ≥3 (OR = 1.51; 95% CI = 1.17–1.94). Alleviating the effects of these factors could potentially decrease the proportion of patients with a disability after a distal radial fracture.

**Level of evidence:** III

## Introduction

Distal radial fracture (DRF) has an incidence of 26 per 10,000 persons per year, making it the commonest fracture in the adult population (Brogren et al., 2007; Court-Brown and Caesar, 2006). In recent decades, there have been changes in the treatment of DRF with a trend towards fixation using an anterior locking plate (Wilcke et al., 2013) with good outcomes as shown by clinical trials (Lundqvist et al., 2022b; Rozental et al., 2009). Despite technical advances and the observed changes in treatment strategy, a population-based study in southern Sweden found that 1-year patient-reported outcome during the period 2003–2012 remained similar and that 17% of all patients included in the study continue to have major disabilities (Disability of the Arm, Shoulder and Hand (DASH) score >35) (Landgren et al., 2017). Other studies have reported a similar proportion with disabilities (MacDermid et al., 2003; Sagerfors et al., 2023). The aim of the surgical treatment is to restore the anatomy, but the radiographic results after surgery for DRF have shown poor correlation with the clinical outcome (Lundqvist et al., 2022b; Schmidt et al., 2024). In addition, the correlation between post-traumatic osteoarthritis and clinical symptoms remains unclear (Goldfarb et al., 2006; Lundqvist et al., 2022a).

Studies have linked psychosocial factors to the outcome after surgery for hand injuries and other conditions affecting the hand and wrist (Goudie et al., 2022; Souer et al., 2008). For hip replacement surgery, moderate to severe problems regarding self-care, anxiety/depression and pain are more common among patients born outside Sweden both before and after surgery (Krupic et al., 2014). Similar studies examining the relationship between psychosocial factors and outcomes in patients with DRF are scarce.

We hypothesized that socioeconomic factors, country of birth, as well as psychiatric comorbidity, are factors associated with poor patient-reported outcome. The aim of this study was to investigate whether somatic or psychiatric comorbidity, socioeconomic factors, and country of birth are associated with a poor outcome 1 year after a DRF.

## Methods

### Study design and patient population

This is a retrospective register-based cohort study with prospectively collected data. The patient population was obtained from the Swedish Fracture Register (SFR). All registrations between 1 April 2012 and 31 December 2018 with an International Classification of Diseases (ICD)-10 code S52.5 or S52.6 in patients 18 years or older were included in the study. Bilateral fractures and physeal plate fractures were excluded. Registrations in the SFR of a DRF in the same patient, side and day were considered duplicate registrations and excluded. Multiple registrations on the same patient during the study period and patients with previous DRFs were not excluded. There were no Swedish language prerequisites for inclusion. Subgroups of this material have been published in previous studies (Sagerfors et al., 2022, 2023).

### Data sources

The SFR is a nation-wide quality register of orthopaedic fractures and their treatment. Inclusion of DRFs in the SFR was started 4 January 2012. At that time, six of 54 orthopaedic departments in Sweden reported to the SFR; in 2018, this number had increased to 45 departments and, at present, all orthopaedic departments in Sweden report to the SFR (Möller et al., 2022). Since 2015, estimates of completeness were made. In 2018, the completeness for ICD-10 codes S52 registrations was 50% and, in 2021, it had reached 69% (Täckningsgradsanalys – Svenska Frakturregistret, n.d.). Patients with a Swedish social security number are eligible for registration in the SFR. At the time of inclusion, data regarding trauma mechanism, fracture classification and treatment were also registered by the physician treating the patient. A letter was sent to the registered patients requesting them to complete the Short Musculoskeletal Function Assessment (SMFA) form reflecting the status before their fracture. Questions regarding smoking habits were also included. One year after the injury, the patients received a letter requesting them to fill out the same questionnaire. In the case of no response after 14 days, a reminder letter was sent once at both the time of injury and one year after. Responses had to be registered within 31 days to be included in the SFR. The letters also contained a stamped return envelope.

The patient population was linked by their social security numbers to Statistics Sweden and The National Board of Health and Welfare where information regarding socioeconomic factors and comorbidities was obtained, respectively. The socioeconomic factors and comorbidity data have been collected by Statistics Sweden and The National Board of Health and Welfare. No consent is needed from the patients regarding the collection of these data. The use of these data for research purposes must be approved by the Swedish Ethical review authority, and the project must thereafter be approved by Statistics Sweden and the National Board of Health and Welfare in Sweden. Our study complied with these standards. The first author (HJ) was responsible for collecting the data for this study, which began in November 2020 and was finished in April 2021.

### Dependent variable

The SMFA is a shortened version of the Musculoskeletal Functional Assessment and consists of 46 questions, 34 of which relate to the function of the patient and 12 relate to how the patient feels about their functional limitations. From the questionnaire, an upper extremity sub-index can be created (Swiontkowski et al., 1999). The Swedish version of SMFA, which has been validated (Ponzer et al., 2003), was used in this study. The dependent variable in our study was poor outcome, which was defined as the quintile of the patients reporting the largest degradation in the SMFA upper extremity (SMFA-UE) subscale 1 year after the fracture compared to the pre-fracture score. The cut-off quantile was based on the outcome information in previous studies with similar patient populations (Landgren et al., 2017; MacDermid et al., 2003).

### Independent variables

In previously unpublished data, we have found that age was not linearly correlated to the log odds of a poor outcome. Age was therefore converted to a categorical variable by quintiles. Type of fracture was classified according to the AO/OTA system, version 2007 (Marsh et al., 2007). The primary types A (extra-articular), B (partial intra-articular) and C (complete intra-articular) were used in the analysis because the reliability of the subtype classification (e.g. A1, A2) has been reported to be poor (Bergvall et al., 2021; Plant et al., 2015). Further, the fracture was also categorized into distal radial or distal radial and distal ulnar fracture (not including ulnar styloid fracture). Trauma mechanism was divided into high-energy and low-energy types. When registering this information in the SFR, the registering physician was given examples of high-energy trauma mechanisms, such as ‘fall from a height’ and ‘traffic accident’. The information regarding treatment method was categorized as surgical or non-surgical. In the SFR, smoking habits were categorized into four groups. This variable was split into two groups: never smoker/previous smoker and daily smoker/sometimes smoker. The country of birth was categorized into three groups: born in Sweden, born in the European Union/Norway/United Kingdom (EU) and born outside the European Union (except Norway/United Kingdom). Marital status was divided into three groups: unmarried, married/registered partnership and divorced/terminated registered partnership/widow(er). Education level was divided into three levels, with low, intermediate and high defined as having <12 years of education (International Standard Classification of Education (ISCED) 0–2), 12 years of education (ISCED 3) and >12 years of education (ISCED 4–8), respectively (OECD, 2015).

Income for the year before the fracture was used in the analysis. It was divided by the price base amount of that year to adjust for inflation. Income was not linearly correlated with poor outcome, based on a logistic regression analysis model including income and a squared version of income as independent variables (data not shown). Thus, income was converted into a categorical variable (low, intermediate and high) according to quantiles (0–25; 26–75; 76–100). Comorbidity was quantified using the Swedish adaptation of the Charlson Comorbidity Index (CCI), which is based on the presence of ICD-10 codes (Ludvigsson et al., 2021). Comorbidity was categorized into four categories (0, 1, 2 or ≥3 diagnoses). Common mental disorders (CMD) of anxiety and depression were defined as the presence of either of the ICD-10 codes F32, F33 or F41 in the Patient Registers, or the presence of the Anatomical Therapeutic Chemical code N06A (antidepressants) in the National Prescribed Drug Register ≥1 year before the fracture.

### Statistical analysis

Continuous variables were reported using median and IQR, and categorical variables were described in terms of frequency and percentage. Multicollinearity among the independent variables was assessed using Pearson’s *r* for continuous variables or Cramér’s *V* for categorical variables (Liebetrau, 1983). Additionally, we calculated standardized differences between responders and non-responders for all independent variables to identify potential selection bias in the responders. The standardized difference was calculated as the difference in the mean or proportion between the two groups, divided by the pooled standard deviation for continuous variables or the squared root of pooled variance for proportion in categorical variables. The absolute value of a standardized difference smaller than 0.1 indicates a negligible difference between the groups, suggesting minimal or negligible selection bias (Hansen and Bowers, 2008).

A multivariate logistic regression analysis was performed. In the first model, each independent variable was tested individually. The second model included all the independent variables exhibiting a statistically significant association in the first model. The results of the analysis were expressed in terms of odds ratio (OR) with the corresponding 95% CI.

To examine effect modification between treatment type and country of birth, an interaction term between treatment and country of birth was added in the second model. Because effect modification was found, a stratified analysis was conducted by applying the second model within each treatment type.

The proportions of missing information in trauma mechanism, treatment method, and smoking were 6.4%, 3.6% and 12.9%, respectively. Therefore, the multiple imputation method was employed to address the missing data with five imputations. The results from the five imputed datasets were then pooled in accordance with Rubin’s rule (Rubin, 2018). A two-sided *p* < 0.05 was considered to be statistically significant. The conduct of the study adhered to the guidelines outlined in the Strengthening the Reporting of Observational Studies in Epidemiology statement (von Elm et al., 2014).

## Results

**Figure 1.**
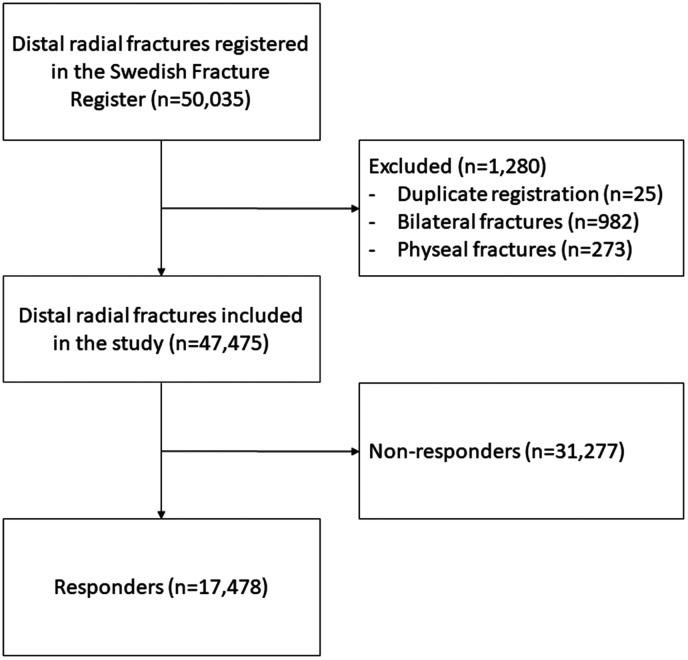
Flow chart of fracture inclusion.

A total of 50,035 DRFs were registered in the SFR during the studied period. Of these, 25 fractures were excluded because of duplicate registration, 273 because of physeal fractures and 982 because of bilateral fractures. Of the remaining 48,755 patients, 17,478 responded to the SMFA questionnaire at inclusion and 1 year after. Figure 1 shows details over patient inclusion. The cut-off value for poor outcome was a SMFA-UE score of 12.5. Table 1 shows the characteristics of the patients included in the study analysis, both for the entire group and grouped according to outcome. The standardized differences between the responders and non-responders are presented in detail in Supplemental Table S1. Multicollinearity analysis showed no correlation coefficient > 0.7. In the first model, all independent variables exhibited statistically significant associations, thus included in the second model. The complete results from the first and second models are presented in Table 2 and Supplemental Figure S1. The interaction term between country of birth and treatment method was statistically significant (*p* = 0.02). The stratified analysis indicated that for patients treated operatively, the OR (95% CI) for patients with country of birth outside EU/UK/Norway and in EU/UK/Norway was 2.65 (2.01–3.49) and 1.12 (0.87–1.43), respectively, with being born in Sweden as a reference. For patients treated non-operatively, the OR (95% CI) for country of birth outside EU/UK/Norway and in EU/UK/Norway was 2.06 (1.61–2.64) and 1.55 (1.29–1.86), respectively, with being born in Sweden as reference.

**Table 1.** Characteristics of the patients included in the study analysis, both for the entire group and grouped according to outcome.

Variable	All (*n* = 17,478)		Not poor outcome (*n* = 14,246)		Poor outcome (*n* = 3,232)	
	*n*	%	*N*	%	*n*	%
Age (years)						
18–49	3460	20	2954	85	506	15
50–60	3701	21	3004	81	697	19
61–68	3359	19	2798	83	561	17
69–77	3479	20	2869	83	610	18
≥78	3479	20	2621	75	858	25
Sex						
Female	14,349	82	11,587	81	2762	19
Male	3129	18	2659	85	470	15
Fracture classification						
AO type A	10,980	63	9045	82	1935	18
AO type B	2026	12	1682	83	344	17
AO type C	4472	26	3519	79	953	21
Fractured bone(s)						
Radius	16,073	92	13,180	82	2893	18
Radius + Ulna	1405	8.0	1066	76	339	24
Trauma mechanism						
Low-energy	15,604	89	12,742	82	2862	18
High-energy	759	4.3	576	76	183	24
Missing	1115	6.4	928	83	187	17
Treatment						
Non-surgical	10,401	60	8609	83	1792	17
Surgical	6450	37	5152	80	1298	20
Missing	627	3.6	485	83	142	17
Country of birth						
Sweden	15,644	90	12,901	83	2743	18
EU/Norway/UK	1168	6.7	886	76	282	24
Outside EU	666	3.8	459	69	207	31
Marital status						
Unmarried	3243	19	2768	85	475	15
Married	8845	51	7333	83	1512	17
Divorced/widow(er)	5380	31	4136	77	1244	23
Missing	10	0.1	9	90	1	10
Education level						
Low	3715	21	2862	77	853	23
Intermediate	7506	43	6026	80	1480	20
High	6166	35	5285	86	881	14
Missing	91	0.5	73	80	18	20
Income level						
Low	3383	19	2663	79	720	21
Intermediate	9191	53	7348	80	1843	20
High	4892	28	4223	86	669	14
Missing	12	0.1	12	100	0	0
Smoking						
No	13,718	79	11,262	82	2456	18
Yes	1497	8.6	1146	77	351	23
Missing	2263	13	1838	81	425	19
CMD						
No	16,346	94	13,445	82	2901	18
Yes	1132	6.5	801	71	331	29
CCI						
0	13,234	76	10,971	83	2263	17
1	3021	17	2362	78	659	22
2	874	5.0	667	76	207	24
≥3	349	2.0	246	71	103	30

Abbreviations: AO: Arbeitsgemeinschaft für Osteosynthesefragen; CCI: Charlson comorbidity index; CMD: common mental disorders; EU: European Union; UK: United Kingdom.

**Table 2.** Logistic regression analysis. In model 1, each independent variable was analysed individually. In model 2, all independent variables were included.

	Model 1				Model 2			
			95% CI limits				95% CI limits	
Variable	*p*-value	OR	Lower	Upper	*p*-value	OR	Lower	Upper
Age (ref 61–68)								
18–49	0.02	0.85	0.75	0.97	0.37	0.94	0.81	1.08
50–60	0.02	1.16	1.02	1.31	0.004	1.20	1.06	1.36
69–77	0.36	1.06	0.94	1.20	0.62	0.97	0.85	1.10
≥78	<0.001	1.63	1.45	1.84	<0.001	1.36	1.19	1.56
Sex (ref female)								
Male	<0.001	0.74	0.67	0.83	<0.001	0.74	0.66	0.83
Fracture classification (ref AO type A)								
AO type B	0.48	0.96	0.84	1.08	0.97	1.00	0.88	1.14
AO type C	<0.001	1.27	1.16	1.38	<.001	1.24	1.13	1.36
Fractured bone(s) (ref radius)								
Radius + ulna	<0.001	1.45	1.27	1.65	0.002	1.23	1.08	1.41
Trauma mechanism (ref low-energy)								
High-energy	<0.001	1.36	1.15	1.61	<0.001	1.76	1.46	2.12
Treatment (ref non-surgical)								
Surgical	<0.001	1.20	1.11	1.30	<0.001	1.20	1.01	1.31
Country of birth (ref Sweden)								
EU/Norway/UK	<0.001	1.50	1.30	1.72	<0.001	1.36	1.18	1.57
Outside EU/Norway/UK	<0.001	2.12	1.79	2.51	<0.001	2.28	1.91	2.73
Marital status (ref Married)								
Unmarried	0.001	0.83	0.74	0.93	0.07	0.89	0.79	1.01
Divorced/widow(er)	<0.001	1.46	1.34	1.59	<0.001	1.24	1.13	1.35
Education level (ref high)								
Low	<0.001	1.79	1.61	1.99	<0.001	1.40	1.24	1.57
Intermediate	<0.001	1.48	1.35	1.62	<0.001	1.35	1.22	1.48
Income level (ref intermediate)								
Low	0.14	1.08	0.98	1.19	0.28	0.94	0.85	1.05
High	<0.001	0.63	0.57	0.70	<0.001	0.79	0.70	0.87
Smoking (ref no)								
Yes	<0.001	1.33	1.17	1.51	<0.001	1.28	1.12	1.46
CMD (ref no)								
yes	<0.001	1.92	1.68	2.19	<0.001	1.46	1.26	1.70
CCI (ref no)								
1	<0.001	1.35	1.23	1.49	0.002	1.18	1.07	1.31
2	<0.001	1.51	1.28	1.77	0.09	1.17	0.98	1.40
≥3	<0.001	2.03	1.61	2.57	0.001	1.51	1.17	1.94

Abbreviations: AO: Arbeitsgemeinschaft für Osteosynthesefragen; CCI: Charlson comorbidity index; CMD: common mental disorders; EU: European Union; OR: odds ratio; ref: reference; UK: United Kingdom.

## Discussion

The aim of this register-based cohort study was to investigate socioeconomic factors associated with the risk of poor patient-reported outcome after a DRF. The most important factors associated with a poor outcome found in this study were country of birth outside EU, high-energy trauma mechanism, CMD and CCI ≥ 3.

To our knowledge, the association between country of birth and outcome after DRF has not been studied previously. Health literacy is the ability to understand and use healthcare information are important (Liu et al., 2020). It has been reported that measures of health literacy are lower among Arabic speaking immigrants in Sweden, especially among those who have spent a short time in Sweden (Bergman et al., 2021). Health literacy and DRF has not been studied, but it is an important factor for the outcome after total knee arthroplasty (Narayanan et al., 2021), and it has a negative impact on musculoskeletal pain treatment and coping. One action to improve health literacy could entail education and information in the patient’s native language. In the analysis stratified according to treatment method, the difference in OR for poor outcome between the patients born in EU and outside EU was more pronounced in the subgroup treated operatively compared to the subgroup treated non-operatively. This suggests that actions are especially needed for patients treated operatively.

Using the same patient population as the present study, we have previously reported that a high-energy trauma mechanism was more common among patients with AO type C3 fractures and that the outcome among patients with AO type C3 was worse compared to patients with AO type C1 and C2 fractures. We did not include AO subclassifications in our regression models because the reliability has been questioned (Plant et al., 2015). It is likely that part of the association between the high-energy trauma mechanism and a poor outcome is explained by the more complex fracture pattern of the AO type C3 fractures. There was a correlation between the multi-trauma and high-energy trauma mechanisms, both of which have been associated with worse Patient-Reported Wrist Evaluation scores after a DRF in a study by Hodel et al. (2020).

The negative effect of psychological factors on DRF outcomes has been established in previous studies (Goudie et al., 2022; Jayakumar et al., 2018, 2020). In our study, CMD was one of the most important factors associated with poor outcome, concurring with previous reports. Ring et al. (2006) have shown that self-reported upper extremity-specific health status as measured with the DASH score correlated with depression and pain anxiety. Psychosocial factors measured early after fracture have also been found to be associated with pain and disability up to 9 months after a DRF (Goudie et al., 2022). These results are in line with our findings. Previous interventions aimed at this patient group include the use of a workbook aimed at optimizing the psychological response to injury, mind body skills based intervention and mindfulness exercise (Goudie et al., 2018; Vranceanu et al., 2015; Westenberg et al., 2018). Further studies focusing on interventions for this patient group are needed.

A CCI ≥ 3 was strongly associated with poor outcome. Increasing CCI is a predictor of non-operative treatment among patients with DRF (Walsh et al., 2022). The association between CCI and poor outcome may be due to a tendency to treat even complex fractures non-operatively in patients with many comorbidities. Lower extremity dysfunction can affect the DASH score (Dowrick et al., 2006). Whether SMFA-UE also exhibits this property is not clear. Thus, residual confounding could be considered as an explanation the association between CCI ≥ 3 and poor outcome.

This study has limitations. The completeness of the SFR is poor, especially in the early years of registration. In 2021, completeness on a national scale was 68% for DRFs. However, this likely represents random error, and its interference with our results is probably minimal. The response percentage was low, which makes our study vulnerable to selection bias. Researchers have investigated the response among non-responders and concluded that the non-responders have similar responses compared to responders (Juto et al., 2017; Stirling et al., 2022). We believe this study has good generalizability, despite the lack of completeness and a low response percentage.

It is likely that the groups with patients born outside Sweden contain fewer patients who have recently immigrated to Sweden and who have a poor knowledge of the Swedish language in comparison to the source population. Given the possible explanation to the association between being born outside Sweden/EU and poor outcome mentioned above, we believe that selection bias may have underestimated the strength of this association in our results. Additionally, some patients may have misinterpreted the questionnaire, potentially affecting the results.

Another limitation is the use of SMFA-UE as an outcome measure. The SMFA-UE has been reported to have a suboptimal measurement error and a noticeable floor effect (a considerable proportion of the participants score at the lower end of the scale) in patients with minor disabilities. The smallest detectable change on the group level is 1.93 (de Graaf et al., 2019). However, the floor effect likely had minimal impact on our results since we used a cut-off score defining poor outcome. We categorized smoking into two categories, non-smoker/previous-smoker and sometimes-smoker/daily smoker. The heterogeneity within the non-smoker/previous-smoker group is acknowledged, but studies suggest a low risk of misclassification regarding the harmful effects of smoking due to substantial benefits of cessation 6 months before surgery (Gräsbeck et al., 2023) and that over 90% of previous smokers report a duration of smoking cessation of at least 2 years (Pesce et al.,2019). The pre-injury SMFA-UE scores were collected up to 1 month after injury, introducing a potential recall bias. The reliability of retrospectively collected patient reported outcome measures in DRF patients is unclear, although it has been deemed feasible in other emergency contexts (Kwong and Black, 2018).

In conclusion, our hypothesis that country of birth, socioeconomic factors and psychiatric comorbidity are associated with poor outcome following a DRF was supported by our findings. Alleviating the effects of these factors could potentially decrease the proportion of patients with disability after a DRF.
